# Hepatic Gene Expression Profiling of American Kestrels (*Falco sparverius*) Exposed In Ovo to Three Alternative Brominated Flame Retardants

**DOI:** 10.3390/biology11091341

**Published:** 2022-09-12

**Authors:** Christopher G. Goodchild, Natalie K. Karouna-Renier, Ryan P. Braham, Paula F. P. Henry, Robert J. Letcher, Kim J. Fernie

**Affiliations:** 1 U.S. Geological Survey Eastern Ecological Science Center—Patuxent Research Refuge, Beltsville, MD 20705, USA; 2 Department of Biology, University of Central Oklahoma, Edmond, OK 73034, USA; 3 U.S. Geological Survey Eastern Ecological Science Center—Leetown Research Laboratory, Kearneysville, WV 25430, USA; 4 U.S. Fish and Wildlife Service Eastern Idaho Field Office, 4425 Burley Drive, Suite A, Chubbuck, ID 83202, USA; 5 U.S. Geological Survey Eastern Ecological Science Center—Patuxent Research Refuge, Laurel, MD 20708, USA; 6 Ecotoxicology and Wildlife Health Division, Environment and Climate Change Canada, National Wildlife Research Centre, Carleton University, Ottawa, ON K1A 0H3, Canada; 7 Ecotoxicology and Wildlife Health Division, Environment and Climate Change Canada, Burlington, ON L7S 1A1, Canada

**Keywords:** gene expression, brominated flame retardants, avian, toxicology, American kestrel

## Abstract

**Simple Summary:**

Brominated flame retardants are added to many consumer products to reduce flammability. While some of these compounds have been or are being phased out due to toxicity concerns, many other substitute or alternative flame retardant chemicals are still in use and being detected in the environment. There is growing evidence that these alternatives exhibit properties and environmental fates similar to those they replaced. However, little information is available on their potential toxic effects in wildlife. Here, the effects of several flame retardants on American kestrel hatchlings at concentrations observed in wild birds, were investigated by examining gene expression changes in the liver. Effects on the immune, thyroid, and other biological pathways were observed, suggesting that birds exposed as developing embryos in the egg can still exhibit effects upon hatching.

**Abstract:**

A number of brominated flame retardants (BFRs) have been reported to interfere with the thyroid signaling pathway and cause oxidative stress in birds, yet the underlying shifts in gene expression associated with these effects remain poorly understood. In this study, we measured hepatic transcriptional responses of 31 genes in American kestrel (*Falco sparverius*) hatchlings following in ovo exposure to one of three high-volume alternative BFRs: 1,2-bis(2,4,6-tribromophenoxy) ethane (BTPBE), bis(2-ethylhexyl)-2,3,4,5-tetrabromophthalate (TBPH), or 2-ethylhexyl-2,3,4,5-tetrabromobenzoate (EHTBB). Hatchling kestrels exhibited shifts in the expression of genes related to oxidative stress (*CYP, GSTA, SOD,* and *GPX1*), thyroid hormone metabolism and transport (*DIO1, DIO2*, and *TTR*), lipid and protein metabolism (PPAR, HMGCR, FAB1, and LPL), and cytokine-mediated inflammation (*TLR3, IL18, IRF7, STAT3, RACK1,* and *CEBPB*). Male and female hatchlings differed in which genes were differentially expressed, as well as the direction of the effect (up- vs. downregulation). These results build upon our previous findings of increased oxidative stress and disrupted thyroid signaling pathway in the same hatchlings. Furthermore, our results indicate that inflammatory responses appear to occur in female hatchlings exposed to BTBPE and EHTBB in ovo. Gene expression analysis revealed multiple affected pathways, adding to the growing evidence that sublethal physiological effects are complex and are a concern for birds exposed to BTBPE, EHTBB, or TBPH in ovo.

## 1. Introduction

The incorporation of alternative brominated flame retardants (BFRs) into consumer products has substantially increased following concerns about the toxicity and biomagnification of established and historically used flame retardant chemicals such as polybrominated diphenyl ethers (PBDEs) [1]. However, like PBDEs, many alternative BFRs are not chemically bonded to their target (polymer) products, and there is growing evidence that alternative BFRs have physicochemical properties and environmental behavior similar to PBDEs [2,3]. For instance, three high-volume alternative BFRs (bis(2-ethylhexyl)-2,3,4,5-tetrabromophthalate (TBPH, also abbreviated BEHTBP), 2-ethylhexyl-2,3,4,5-tetrabromobenzoate (EHTBB), and 1,2-bis(2,4,6-tribromophenoxy) ethane (BTPBE)) have been detected in leachate from landfills [4].

Long-range atmospheric transport has led to TBPH, EHTBB, and BTBPE being present in soil and water samples, as well as aquatic biota collected from remote locations [5,6,7,8]. These three BFRs have also been detected in the liver, kidney, and muscle tissues from predatory birds and shorebirds, indicating trophic transfer of these alternative BFRs [9,10,11,12]. Additionally, there is evidence that BTBPE, EHTBB, and TBPH are maternally transferred from mother birds to their eggs. Brünnich’s guillemot (*Uria lomvia*) eggs collected from the Norwegian Arctic had detectable levels of EHTBB (mean 11 ng/g lipid wt) and TBPH (19 ng/g lipid wt) [13]. Several studies have also detected BTBPE in the eggs of shorebirds, though BTBPE appears to be more readily metabolized and tends to be present at lower concentrations (range: 0.1–2 ng/g wet wt) [13,14,15,16]. In addition to being present in shorebirds and ducks, BTBPH, EHTBB, and TBPH have also been detected in raptors. Peregrine falcon (*F. peregrinus*) eggs collected from the low Arctic in South Greenland contained TBPH (range: <0.25–2.38 ng/g lipid wt), EHTBB (range: <0.62–54.3 ng/g lipid wt), and BTBPE (range: <0.83–11.8 ng/g lipid wt) [17,18,19,20]. Given the prevalence of BTBPH, EHTBB, and TBPH in avian eggs, concerns have been raised about the potential for in ovo exposure to these alternative BFRs to cause embryotoxicity.

The American kestrel (*Falco sparverius*) is an apex predator and an important toxicological model for predatory birds [21] with relatively high sensitivity to environmental contaminants. We previously reported disruption of thyroid function and increased oxidative stress in American kestrel hatchlings exposed in ovo to environmentally relevant concentrations of BTBPE, EHTBB, and TBPH [22,23]. Specifically, kestrel hatchlings exposed to EHTBB or TBPH in ovo (range: nominal concentrations 10–100 ng/g egg mass) exhibited lower thyroidal colloid (EHTBB and TBPH), as well as higher plasma triiodothyronine (T3) and lower glandular thyroxine (T4) (EHTBB only) [23]. Additionally, kestrel hatchlings exposed to BTBPE, EHTBB, or TBPH in ovo exhibited altered activity of hepatic deiodinases, enzymes that convert T4 to the biologically active form T3, but the effects of these alternative BFRs on deiodinase activity varied by sex [22,23]. In addition to dysregulation of thyroid signaling, in ovo exposure to BTBPE, EHTBB, and TBPH increases oxidative stress in hatchlings. Kestrel hatchlings exposed to EHTBB and TBPH in ovo exhibited higher glutathione levels [23]. While these results indicate that in ovo exposure to BTBPE, EHTBB, and TBPH increases oxidative stress and interferes with several components of the thyroid signaling pathway in kestrel hatchlings, the transcriptional responses to in ovo exposure to these alternative BFRs are unknown.

“Omics” approaches (e.g., transcriptomics, toxicogenomics, and metabolomics) provide insights into pathway perturbations and can identify potential (eco)toxicological mechanisms of action by measuring differentially expressed genes in response to environmental chemicals [24]. In the present study, we measured hepatic transcript abundance of 31 genes in the same American kestrel hatchlings exposed to EHTBB, TBPH, or BTBPE in ovo, in which we previously reported increased oxidative stress and thyroid system dysregulation using nongenomic biomarkers [22,23]. Given this previous evidence, we hypothesized that in ovo exposure to BTBPE, EHTBB, and TBPH would affect the mRNA expression of detoxification enzymes and genes related to thyroid signaling, and that these changes would be sex-specific. However, our objectives for this study also included the exploration/identification of other pathways/genes that may be affected by exposure to these chemicals, to better understand the potential hazards of these flame retardants and their toxicological effects on birds. Therefore, we examined the expression of genes from a range of toxicologically relevant pathways including the thyroid axis, xenobiotic metabolism, lipid homeostasis, oxidative stress, immune function, and the PPAR signaling pathway [25] (Table 1).

## 2. Materials and Methods

### 2.1. Exposures

The US Geological Survey Eastern Ecological Science Center (EESC) Animal Care and Use Committee (2006-06) reviewed and approved all procedures and protocols involving live animals. Forty-three breeding pairs from a captive colony of American kestrels maintained at the EESC served as the source of eggs for the study. Eggs (*n* = 205) were collected over 3 weeks and temporarily stored, as necessary, for up to 7 days at 13 °C to allow for synchronization of injections across six batches. Embryo viability was assessed throughout incubation. Eggs were injected into the air cell with 1 μL/g egg (mean weight per batch) of organic safflower oil (control; President’s Choice^®^ Organics) or 10, 50, or 100 (nominal) ng/g egg BTBPE (CAS 37853-59-1, Sigma-Aldrich, St. Louis, MO, USA), TBPH (CAS 26040-51-7, Wellington Laboratories, Guelph, ON, Canada), or EHTBB (CAS 183658-27-7, Wellington Laboratories) dissolved in safflower oil. Eggs from each parental pair were included in the four treatment groups. Eggs were incubated horizontally at 37.5 °C and rotated 180° every hour [26,27] and then transferred to a hatcher unit on embryonic day (ED) 24 until hatching. Hatchlings were allowed to dry and were euthanized by decapitation within 24 h of hatching. Necropsies were performed immediately, and livers were collected for analysis. Egg incubation and exposure specifics are provided in Guigueno et al. [28] and Eng et al. [22]. Genetic sex was determined as described in Eng et al. [22].

### 2.2. Dosing Solutions and Chemical Analysis

Preparation of dosing solutions was described previously [23]. Gas chromatography–single-quadrupole mass spectrometry operating in the electron-capture negative ionization mode was used for analysis of dosing solutions [22,28]. Measured concentrations were 5, 27, and 58 ng BTBPE/μL, 11, 55, and 137 ng EHTBB/µL, and 12, 60, and 107 ng TBPH/µL. The concentrations of BTBPE, EHTBB, and TBPH in the yolk sac and liver of these same kestrel hatchlings were previously reported [22,23].

### 2.3. Gene Expression Analysis

Total RNA was extracted from liver using RNAzol^®^ RT (Molecular Research Center, Inc., Cincinnati, OH, USA) following the manufacturer’s protocol. RNA concentration was determined on a Qubit4 using the RNA BR Assay Kit (Thermo-Fisher Scientific, Agawam, MA, USA). Six biological replicates were analyzed per dose for each sex, except for 50 and 100 ng/g EHTBB females (*n* = 5 and 4, respectively), 50 ng/g EHTBB males (*n* = 5), and 100 mg/g BTBPE males (*n* = 5). Transcript abundance of 31 genes (Table 1) associated with xenobiotic metabolism, immune function, the PPAR signaling pathway, lipid homeostasis, thyroid hormone metabolism and transport, and oxidative stress was analyzed using the NanoString nCounter SPRINT™ (NanoString Technologies, Seattle, WA, USA). Three housekeeping genes (Table 1) and positive and negative controls were used for normalization (see Statistical Analysis). The custom CodeSet was designed by NanoString Technologies (Seattle, WA, USA) based on sequences selected a priori from a previously assembled American kestrel de novo transcriptome (Karouna-Renier, unpublished data; Accession ID: PRJNA313951).

### 2.4. Statistical Analysis

Prior to conducting pairwise comparisons of differential gene expression, we first tested whether there were overall sex-specific differences in gene expression in males and females exposed to BTBPE, EHTBB, and TBPH. To do so, we used the NanoStringDiff package (Version 1.20.0) [29] in R (Version 4.0.2; R Core Team) to normalize transcript abundance data, whereby size factors were first calculated from positive controls and housekeeping controls, and background level was obtained from negative controls. We then calculated the relative difference of transcript abundance in hatchlings exposed to BTBPE, EHTBB, and TBPH compared to sex-specific controls for each individual and target gene. We conducted three separate principal component analyses (PCAs) using the ‘prcomp’ function in R (Version 4.0.2), each of which included only individuals exposed to BTBPE, EHTBB, or TBPH. In the first PCA, we tested for overall sex differences in transcript abundance among hatchlings but did not examine differences among concentrations within each BFR (i.e., levels within BTBPE, EHTBB, and TBPH were pooled). Following this analysis, we conducted separate PCAs for males and females to assess whether different concentrations of BTBPE, EHTBB, and TBPH led to changes in transcript abundance.

Additionally, we analyzed differential expression of individual genes for males and females separately using the NanoStringDiff package. This package facilitates pairwise comparisons by utilizing a generalized linear model (GLM) of the negative binomial family to characterize count data, and it was designed specifically for transcript abundance data. Transcript abundance was normalized as previously described, and then controls were incorporated into the model framework to utilize all data points [29]. This method uses an empirical Bayes shrinkage approach to estimate the dispersion parameter and a likelihood ratio test to identify differential expression of genes, with the Benjamini–Hochberg procedure (false discovery rate threshold = 0.1) [29]. The differential expression (log_2_ fold change) of each gene was compared to sex-specific controls at alpha = 0.10. Because of the exploratory nature of this study and so as not to overlook potential perturbations to biochemical pathways, we chose a less conservative alpha for identification of differentially expressed genes. Genes with *p* ≤ 0.10 were considered to be of potential biological interest (up- or downregulated), and genes with *p >* 0.10 were considered unchanged. Tables S1 and S2 provide exact *p*-values for each gene/dose group.

## 3. Results

**Figure 1:**
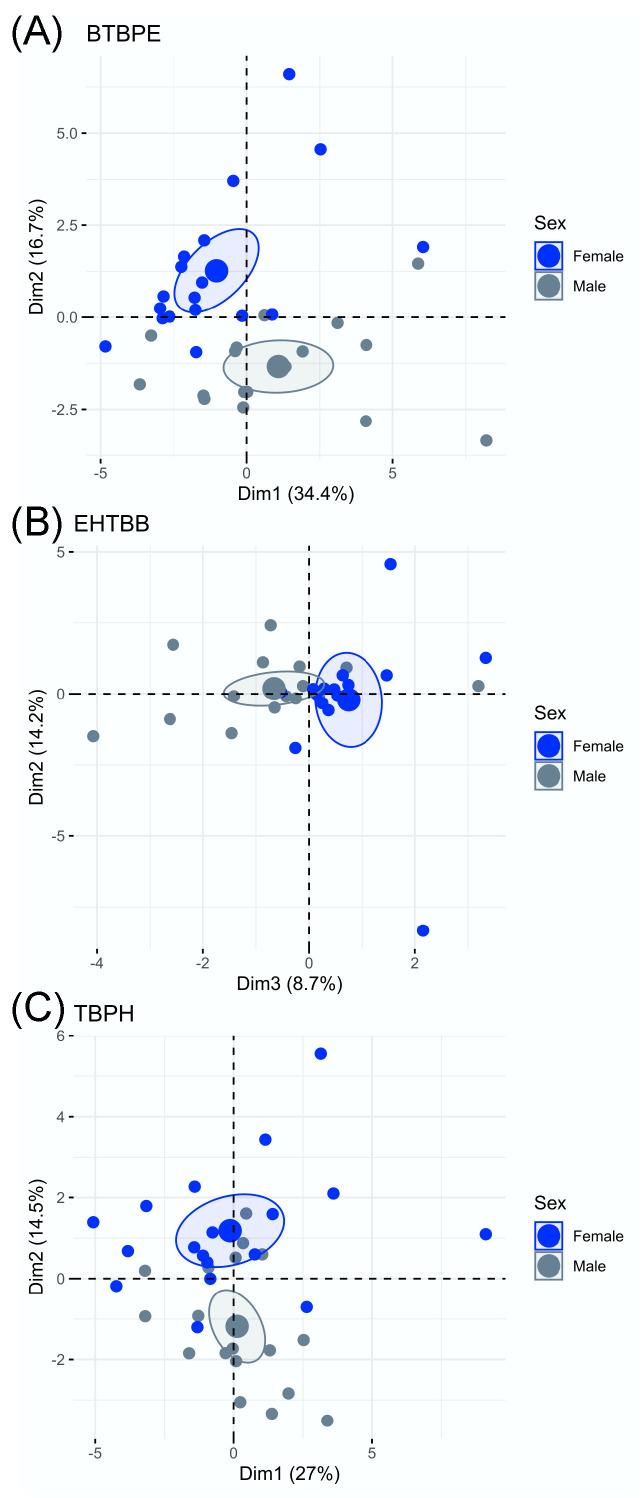
PCA of gene expression in male (gray) and female (blue) American kestrel (*Falco sparverius*) hatchlings exposed in ovo to BTBPE (**A**), EHTBB (**B**), or TBPH (**C**) relative to sex-specific controls. Small points represent individual hatchlings, large points denote mean coordinates for each sex, and ellipses denote the 95% confidence interval for group mean coordinates.

**Figure 2:**
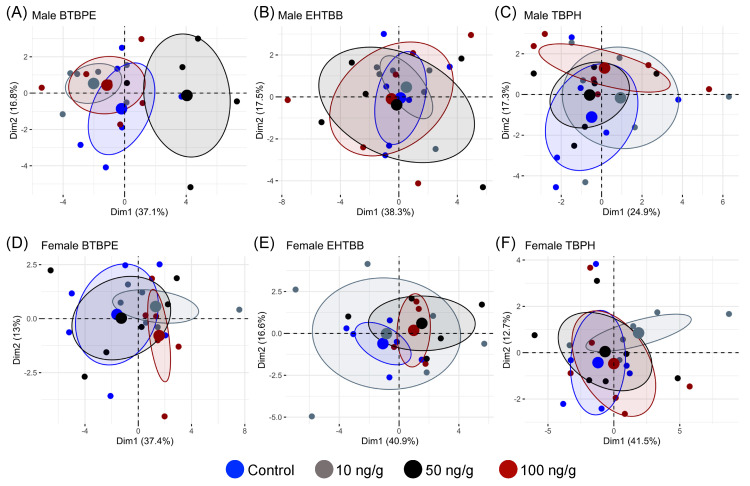
PCA from hepatic transcript abundance of male (**A**–**C**) and female (**D**–**F**) American kestrels (*Falco sparverius)* exposed in ovo to BTBPE, EHTBB, or TBPH. Small points represent individual hatchlings, large points denote mean coordinates of each concentration, and ellipses denote the 95% confidence intervals for group mean coordinates.

Male and female hatchlings exhibited different transcriptional responses to in ovo exposure to BTBPE, EHTBB, and TBPH (Figure 1). We observed differential expression of genes related to (1) xenobiotic metabolism and oxidative stress, (2) thyroid hormone metabolism and transport, (3) lipid and protein metabolism, and (4) cytokine-mediated inflammation. In most cases, the log_2_ fold change in transcript abundance was <1.0 for differentially expressed genes (*p* < 0.10). Five of the gene targets exhibited low counts (*FABP4, IFNB, IFNG, IL6*, and *RIGI*), and their data were not included in the analyses. Although hatchlings exposed to BTBPE, EHTBB, and TBPH exhibited several differentially expressed genes, the PCA of transcript abundance for each sex indicated there were no overall differences in global gene expression between treatments (Figure 2).

### 3.1. Expression of Genes Related to Xenobiotic Metabolism and Oxidative Stress

**Figure 3:**
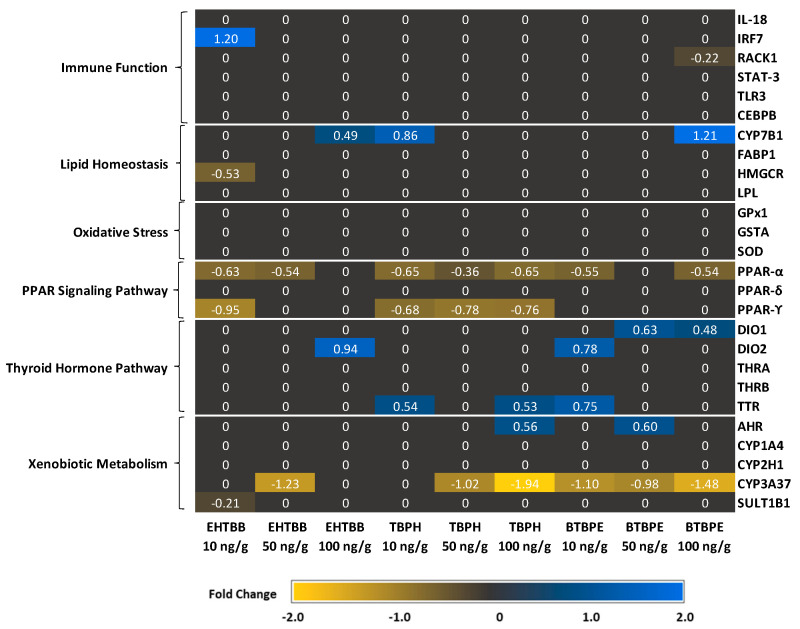
Differential expression of mRNA transcripts of 26 genes in male American kestrel (*Falco sparverius)* hatchling livers analyzed using a Nanostring codeset following in ovo exposure to 10, 50, and 100 ng/g of the flame retardants EHTBB, TBPH, and BTBPE. The heat map indicates the mean log_2_ fold change for each dose group. Genes with *p* ≤ 0.10 are identified in blue (upregulation) or orange (downregulation). Genes with *p >* 0.10 were set to 0 to minimize noise and are shown in black.

**Figure 4:**
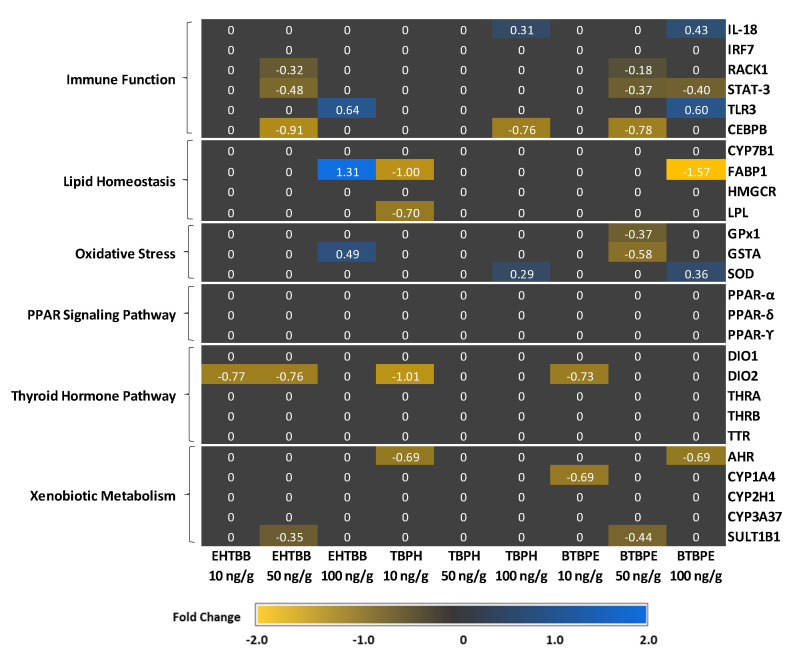
Differential expression of mRNA transcripts of 26 genes in female American kestrel (*Falco sparverius)* hatchling livers analyzed using a Nanostring codeset following in ovo exposure to 10, 50, and 100 ng/g of the flame retardants EHTBB, TBPH, and BTBPE. The heat map indicates the mean log_2_ fold change for each dose group. Genes with *p* ≤ 0.10 are identified in blue (upregulation) or orange (downregulation). Genes with *p >* 0.10 were set to 0 to minimize noise and are shown in black.

Exposure to BTBPE, EHTBB, and TBPH altered the expression of genes in the cytochrome P450 (CYP) gene family. *CYP3A37* was downregulated in male hatchlings exposed to 10, 50, and 100 ng/g BTBPE, 50 ng/g EHTBB, or 50 and 100 ng/g TBPH (Figure 3). *CYP1A4* was the only gene in the CYP family to be differentially expressed in female hatchlings and was downregulated in female hatchlings exposed to 10 ng/g BTBPE but was unaffected in females exposed to EHTBB and TBPH or males exposed to any treatment (Figure 3 and Figure 4).

Expression of the sulfotransferase enzyme *SULT1B1* was downregulated in both male (10 ng/g) and female (50 ng/g) hatchlings exposed to EHTBB but the fold change was <1.0 (Figure 3 and Figure 4). Additionally, *SULT1B1* was downregulated in female hatchlings exposed to 50 ng/g BTBPE, but not in female hatchlings exposed to 10 or 100 ng/g BTBPE, nor in males exposed to any concentration of BTBPE (Figure 3 and Figure 4). Exposure to TBPH did not influence *SULT1B1* transcript abundance in males or females.

Exposure to BTBPE and TBPH increased expression of aryl hydrocarbon receptor (*AHR*) in male hatchlings but decreased *AHR* expression in female hatchlings. Specifically, *AHR* expression was upregulated in male hatchlings exposed to 50 ng/g BTBPE and 100 ng/g TBPH (Figure 3) and downregulated in female hatchlings exposed to 100 ng/g BTBPE and 10 ng/g TBPH (Figure 4). No other effects were observed on *AHR* expression in female or male hatchlings. *AHR* was not differentially expressed in male or female hatchlings exposed to EHTBB.

Exposure to BTBPE, EHTBB, and TBPH in ovo affected the expression of genes involved in the glutathione detoxification pathway and superoxide dismutase (*SOD*) expression, but these transcriptional responses only occurred in females (Figure 4). Female hatchlings exposed to 50 ng/g BTBPE exhibited lower glutathione *S*-transferase (*GSTA*) and glutathione peroxidase (*GPX1*) expression compared to female controls (Figure 3), but similar trends did not occur in female hatchlings exposed to 10 or 100 ng/g BTBPE. Neither EHTBB nor TBPH affected *GPX1* expression. *GSTA* was upregulated in female hatchlings exposed to 100 ng/g EHTBB (Figure 4), but similar trends did not occur in female hatchlings exposed to 10 or 50 ng/g EHTBB nor any concentration of TBPH. *SOD* was upregulated in female hatchlings exposed to 100 ng/g BTBPE and 100 ng/g TBPH (Figure 4), but not in those exposed to any concentration of EHTBB.

### 3.2. Expression of Genes Related to Thyroid Metabolism and Transport

Both male and female hatchlings exposed to BTBPE, EHTBB, and TBPH exhibited differential expression of deiodinase (DIO) genes, but males tended to have increased DIO expression, whereas females tended to have lower DIO expression (Figure 3 and Figure 4). *DIO1* expression was upregulated in male hatchlings exposed to 50 ng/g or 100 ng/g BTBPE (Figure 3) but was unaffected upon exposure to EHTBB or TBPH (*p >* 0.10). There were no differences in *DIO1* expression in female hatchlings exposed to TBPH, BTBPE, or EHTBB. In ovo exposure to 10 ng/g BTBPE and 100 ng/g EHTBB resulted in higher *DIO2* expression in male hatchlings compared to control males (Figure 3). Conversely, *DIO2* was downregulated in female hatchlings exposed to 10 ng/g BTBPE, 10 ng/g TBPH, and 10 ng/g and 50 ng/g EHTBB (Figure 4).

Exposure to BTBPE and TBPH also increased expression of the thyroid hormone chaperone protein, transthyretin (*TTR*), in male hatchlings (Figure 3), but *TTR* was not differentially expressed in female hatchlings. Male hatchlings exposed to 10 ng/g BTBPE, 10 ng/g TBPH, or 100 ng/g TBPH exhibited higher expression of *TTR* compared to male controls (Figure 3). *TTR* was not differentially expressed in male hatchlings exposed to any concentration of EHTBB.

### 3.3. Expression of Genes Related to Lipid and Protein Metabolism

Several genes related to energy metabolism and protein synthesis were differentially expressed, and responses often differed between males and females (Figure 3 and Figure 4). Male hatchlings exposed to BTBPE, EHTBB, and TBPH exhibited lower expression of the peroxisome proliferator-activated receptors (PPAR), but PPAR receptors were not differentially expressed in female hatchlings. *PPARA* expression was downregulated in all male hatchlings, although two exposures (100 ng/g EHTBB and 50 ng/g BTBPE) exhibited *p >* 0.10. *PPARG* expression was lower in male hatchlings exposed to 10 ng/g EHTBB and all concentrations of TBPH (Figure 3).

Exposure to BTBPE, EHTBB, and TBPH also influenced the expression of several genes related to cholesterol metabolism and protein synthesis. Expression of CYP7B1, which encodes the cholesterol and bile acid intermediate-metabolizing enzyme oxysterol 7-alpha-hydroxylase, was upregulated in male hatchlings exposed to 10 ng/g TBPH, 100 ng/g EHTBB, and 100 ng/g BTBPE (Figure 3). *CYP7B1* expression was not altered in female hatchlings. Expression of the rate-controlling enzyme in the cholesterol synthesis pathway, 3-hydroxy-3-methyl-glutaryl-coenzyme A reductase (*HMGCR*), tended to be lower in male hatchlings exposed to EHTBB treatments (Figure 3). Neither BTBPE nor TBPH affected *HMGCR* expression in males, nor did any treatment alter *HMGCR* expression in females.

Fatty acid-binding protein (*FABP1*) was differentially expressed in females exposed to BTBPE, EHTBB, and TBPH, but not in males. Specifically, FABP1 was upregulated in female hatchlings exposed to 100 ng/g EHTBB but downregulated in female hatchlings exposed to 100 ng/g BTBPE (Figure 4). *FABP1* and lipoprotein lipase (*LPL*) were also downregulated in female hatchlings exposed to 10 ng/g TBPH (Figure 4). *LPL* expression was not differentially expressed in male or female hatchlings exposed to any other treatment (Figure 3 and Figure 4).

### 3.4. Expression of Immune System-Related Genes

Several genes involved in immune function and inflammation were differentially expressed in female hatchlings (Figure 4). Expression of the proinflammatory cytokine interleukin, *IL18,* was upregulated in female hatchlings exposed to 100 ng/g BTBPE and 100 ng/g TBPH. *IL18* was not differentially expressed in female hatchlings exposed to 10 or 50 ng/g of BTBPE or TBPH, nor female hatchlings exposed to any concentration of EHTBB. Exposure of female hatchlings to BTPBE and EHTBB also affected the expression of transcription factors involved in cytokine-mediated proinflammatory responses. The transcription factor signal transducer and activator of transcription 3 (*STAT3*) was downregulated in females exposed to 50 ng/g BTBPE and 100 ng/g BTBPE (Figure 4) but not 10 ng/g BTBPE. Additionally, *STAT3* was downregulated in female hatchlings exposed in ovo to 50 ng/g EHTBB (Figure 4), but not 10 or 100 ng/g EHTBB. *STAT3* was not differentially expressed in hatchlings exposed to any concentration of TBPH or in male hatchlings.

The transcription factor interferon regulatory factor 7 (*IRF7*) was upregulated in male hatchlings exposed to 10 ng/g EHTBB (Figure 3). Expression of the scaffolding protein receptor for activated C kinase 1 (*RACK1*) was lower male hatchlings exposed 100 ng/g BTBPE (Figure 3) and female hatchlings exposed to 50 ng/g BTBPE (Figure 4). Additionally, *RACK1* expression was lower in female hatchlings exposed to 50 ng/g EHTBB (Figure 4). *RACK1* was not differentially expressed in males exposed to EHTBB nor males and females exposed to any concentration of TBPH.

The transcription factor CCAAT/enhancer-binding protein beta (*CEBPB*) was downregulated in female hatchlings exposed to 50 ng/g EHTBB (Figure 4). *CEBPB* was not differentially expressed in male nor female hatchlings exposed in ovo to any concentration of BTBPE or TBPH. Expression of Toll-like receptor 3 (*TLR3*), which is involved in innate immunity, was upregulated in female hatchlings exposed to 100 ng/g BTBPE and 100 ng/g EHTBB (Figure 4). However, *TLR3* was not differentially expressed in females exposed to other treatments nor in males.

## 4. Discussion

American kestrel hatchlings exposed in ovo to BTBPE, EHTBB, and TBPH exhibited hepatic transcriptional responses indicative of shifts in thyroid function (*DIO1*, *DIO2*, and *TTR*) and oxidative stress (*CYP, SOD, GSTA*, and *GPX1*). These results are generally consistent with previous evidence that in ovo exposure to BTBPE, EHTBB, and TBPH can alter thyroid hormone levels and/or increase oxidative stress [22,23]. In addition to thyroid signaling and oxidative stress, we detected shifts in genes related to energy metabolism (PPAR, I and *LPL*) and immune function (*IL18, IRF7, STAT3, CEBPB, TLR*, and *RACK1*). Furthermore, male and female hatchlings differed in which genes were differentially expressed, as well as the direction of the effect (up- vs. downregulation), which underscores the importance of considering sex-specific effects of in ovo exposure to alternative BFRs and other environmental pollutants.

### 4.1. Effects on Genes Related to Thyroid Function

Differential expression of hepatic *DIO1, DIO2,* and *TTR* indicates alteration in the conversion of T4 to T3 and transport of thyroid hormones. Male hatchlings exposed to BTBPE and EHTBB exhibited higher expression of DIO genes, whereas female hatchlings exhibited lower DIO expression. These transcriptional responses are consistent with previous evidence for sex-specific differences in hepatic deiodinase enzyme activity. That is, we previously observed greater hepatic deiodinase enzyme activity in these same male American kestrel hatchlings exposed in ovo to BTBPE or EHTBB, whereas deiodinase enzyme activity decreased in female hatchlings [22,23]. Exposure to BTBPE and TBPH also led to upregulation of *TTR* transcripts in male hatchlings, but not female hatchlings. Whether these transcriptional effects are compensatory mechanisms in response to a reduction in thyroid synthesis and transport to target tissues or whether in ovo exposure to BTBPE, EHTBB, and TBPH causes over stimulation of the thyroid system is unclear. There is some evidence that BTBPE and EHTBB inhibit deiodinase enzyme activity [30], and studies in glaucus (*Larus hyperboreus*) and herring gulls (*L. argentatus*) suggest that flame retardants may competitively bind to transthyretin [31,32]. Therefore, it is possible that upregulation of DIO genes and TTR in male kestrel hatchlings may be a compensatory response to counteract inhibition of deiodinase activity and lower levels of transthyretin. Alternatively, upregulation of *DIO1, DIO2*, and *TTR* in kestrel males could be the result of a stimulatory effect of BTBPE, EHTBB, and TBPH. We previously reported that these same hatchings exhibited increased thyroidal epithelial cell height to colloid diameter ratio (ECH:CD), suggesting that these chemicals had a stimulatory effect on the thyroid gland. As others have noted [33], in vitro BFR–TTR competitive binding assays would be a critical initial step toward understanding interactions between alternative BFRs and transthyretin. The reasons for sex-specific differences in DIO and *TTR* responses are not clear, and further research is needed to understand the toxicodynamic pathways driving these differences to better understand the potential ecological hazard posed by BTBPE, EHTBB, and TBPH.

### 4.2. Effects on Genes Related to Xenobiotic Metabolism and Oxidative Stress

Hatchlings exposed to BTBPE, EHTBB, and TBPH exhibited transcriptional responses related to xenobiotic metabolism and oxidative stress, but males and females tended to differ in which genes were differentially expressed. Avian CYP3A37 aids in xenobiotic, cholesterol, and bile acid metabolism [34,35]; in male hatchlings exposed to BTBPE, EHTBB, and TBPH, we observed downregulation of *CYP3A37* expression. This downregulation commonly occurs in response to excessive reactive oxygen species (ROS) generated by phase I metabolism, to prevent oxidative damage [36,37,38]. However, indicators of oxidative stress responses in the male hatchlings, i.e., gene expression (SOD, *GPX1*, and *GSTA*) monitored here and previously reported hepatic biomarkers [22,23], did not indicate altered oxidative status in the liver except at 50 ng/g EHTBB and 100 ng/g TBPH. Expression of hepatic CYP enzymes is also downregulated in response to inflammation or infection [39]. Although we observed limited changes in hepatic immune genes in male hatchlings, the selected immune-related genes may not adequately reflect the actual inflammatory process, if any, caused by exposure to these concentrations. The molecular mechanisms for regulation of *CYP3A7* gene expression are poorly understood; therefore, the causes and potential downstream effects of the transcriptional changes in *CYP3A7* in these birds are unclear. Studies using chicken (*Gallus gallus*), observed no effects on *CYP3A37* expression in hatchlings exposed in ovo to 0.1, 1, and 10 μg/g BTBPE or in chicken hepatocytes exposed in vitro to BTBPE or TBPH (BEHTBP) [33], although exposure to other flame retardants was shown to increase expression [33,40,41]. A comparison of the effects of various flame retardants on CYP expression underscores that some avian models (e.g., chicken) may not be representative of the effects of alternative BFRs on wild birds, and that different alternative BFRs likely vary in their mechanisms of action and toxicity across avian species.

In general, CYP genes were not differentially expressed in female kestrels exposed to BTBPE, EHTBB, or TBPH. However, mRNA levels of the phase I detoxification enzyme SOD were upregulated in female hatchlings exposed to 100 ng/g BTBPE and TBPH. An increase in transcript abundance of this antioxidant enzyme generally supports previous results indicating that these American kestrel hatchlings exposed to TBPH in ovo exhibited alterations in oxidative stress indicators [23]. SOD is the first line of defense against ROS and can be rapidly induced in response to increased levels of oxygen radicals [42]. Although SOD gene expression and enzyme levels are not necessarily synchronized due to post-transcriptional controls, the increased transcription is likely part of the compensatory mechanism that functions to restore redox homeostasis. Increased mobilization or synthesis of multiple components of the antioxidants system functions to physiologically compensate for moderate exposure to ROS [43]. Interestingly, the phase II detoxification enzyme gene, GSTA, was both upregulated (EHTBB) and downregulated (BTBPE) in females, demonstrating the fluidity and complexity of the mechanisms involved in the response to ROS. Increased and sustained oxidative stress can have adverse outcomes for embryonic and nestling development [44]. Although, at the time of sampling, indicators in the liver suggest low levels of oxidative stress, whether or not the examined flame retardants had greater effects earlier in the exposure period is unclear. Therefore, future studies are needed to examine temporal changes to better understand the long-term consequences of oxidative stress for development in predatory birds exposed to BTBPE, EHTBB, and TBPH in ovo.

### 4.3. Effects on Genes Related to Lipid and Protein Metabolism

Several genes related to lipid and protein metabolism were differentially expressed in male and female kestrels exposed to BTBPE, EHTBB, and TBPH. Although males and females differed in which genes were differentially expressed, there was a consistent trend of in ovo exposure to BTBPE, EHTBB, and TBPH causing disruption of genes associated with protein and lipid metabolism, which may have implications for cellular energy homeostasis. For instance, *CYP7B1* was upregulated in male hatchlings exposed to EHTBB and BTBPE. Less is known about the function of CYP7B1 in birds; however, in mammals, it is primarily involved in bile acid synthesis from cholesterol in the liver [45]. CYP7B1 activity and mRNA expression are known to increase in response to higher cholesterol in rodents [45]. However, HMGCR, the rate-limiting gene in cholesterol synthesis, was downregulated in male kestrel hatchlings exposed to EHTBB in the present study. HMGCR is regulated via a negative feedback mechanism mediated by cholesterol and other metabolites [46] and our results suggest that the upregulation of *CYP7B1* and downregulation of *HMGCR* may be a tandem response to reduce cholesterol levels. Previous studies have found increased bile acid synthesis in chicken hatchlings exposed in ovo to multiple chlorinated flame retardants (e.g., TMPP, TDCPP, and TEP; see references for acronyms) [33,41,47]. Although we did not measure hepatic or circulating bile acid or cholesterol concentrations in the present study, together, these data suggest that exposure to brominated and chlorinated flame retardants is potentially altering lipid homeostasis in the hatchling male kestrels.

Additional evidence for effects on lipid homeostasis comes from PPAR genes, which were downregulated in male kestrels exposed to BTBPE, EHTBB, and TBPH. PPARs are transcriptional regulators of lipid metabolism and adipocyte proliferation and differentiation, and they serve as a link between lipid metabolism and the immune system [48]. Fatty acid oxidation (FAO) has anti-inflammatory effects in mammals, and FAO-related genes can be activated by PPAR-α [48]. Downregulation of PPAR-, impairs FAO and can increase lipid availability for adipose tissue accretion and protein synthesis, which are critical for maintaining hatchling growth [49]. PPAR-γ mainly targets fatty acid uptake and storage and regulates cholesterol homeostasis in the liver, the main lipogenic site in birds [50]. Upregulation of *PPARG* in chicken liver is correlated with body fat deposition [50], and, although the effects of hepatic downregulation in birds have not been fully explored, decreased expression of *PPARG* may prevent lipid accumulation in the liver [48]. Our results contrasted with those reported by Guo et al. [51] in adult zebrafish; after 2 weeks of TBPH exposure, expression of PPAR genes increased. Further research is needed to determine whether the transcriptional changes observed in the present study correspond to functional changes in cholesterol, bile acid, and/or fatty acid metabolism that can lead to liver pathology or whether these low-level changes are compensatory in nature.

Although *CYP7B1*, PPAR genes, and *HMGCR* were not differentially expressed in female kestrels, we did observe some shifts in genes related to lipid metabolism in females. Females exposed to BTBPE and TBPH in ovo exhibited lower expression of the fatty acid-binding protein (*FABPl*), as well as decreased expression of *LPL* (TBPH only). FABP1 plays a role in lipid metabolism and transport, and expression levels have previously been reported to decrease in chickens upon exposure to other BFRs [52] and in rainbow trout (*Oncorhynchus mykiss*) exposed to EHTBB and TBPH [53]. Although these transcriptional responses indicate that in ovo exposure to BTBPE, EHTBB, and TBPH may affect hepatic lipid metabolism in both female and male kestrels, further research is needed to understand the functional significance and whether downregulation of these genes impairs energy metabolism.

### 4.4. Effect on Genes Related to Immune Function

In addition to their roles in lipid homeostasis, *PPARA* and *PPARG* expression is known to suppress inflammation by downregulating the expression of proinflammatory genes and upregulating genes with anti-inflammatory properties [48]. Coincidentally with the downregulation of PPARs in males, we observed few changes in the expression of the immune-related genes. However, female hatchlings exhibited several transcriptional responses indicative of cytokine-mediated inflammation. Exposure to BTBPE and EHTBB stimulated proinflammatory pathways, while inhibiting anti-inflammatory pathways. Expression of *TLR*, a gene that regulates transcription of cytokines, increased in females exposed to BTBPE or EHTBB. Upregulation of TLR corresponded with increased expression of the proinflammatory cytokine *IL18* in female hatchlings exposed to BTBPE. Additionally, expression of transcription factor *STAT3*, which is activated by the anti-inflammatory cytokine *IL6*, was downregulated in females exposed to BTBPE or EHTBB. Downregulation of the transcription factor CEBPB, which is activated by both pro- and anti-inflammatory cytokines, in female hatchlings exposed to EHTBB in ovo provides further evidence for cytokine-mediated inflammation. Given that several genes related to hepatic inflammation were differentially expressed, further research is needed to understand whether in ovo exposure to BTBPE and EHTBB can lead to inflammatory liver damage for hatchlings.

## 5. Conclusions

American kestrel hatchlings exposed to environmentally relevant concentrations of BTBPE, EHTBB, or TBPH in ovo exhibited transcriptional responses across several pathways. Genes related to oxidative stress, thyroid signaling pathways, protein and lipid synthesis, and inflammation were differentially expressed, and the responses were often sex-specific. These results build upon previous studies indicating that in ovo exposure to BTBPE, EHTBB, and TBPH increases oxidative stress and alters thyroid function. Furthermore, our results indicate that inflammatory responses occur in females exposed to BTBPE and EHTBB. Although alternative BFRs are not as toxic as PBDEs, differential expression of several transcriptional pathways indicates that sublethal physiological effects are a concern for predatory birds exposed to BTBPE, EHTBB, or TBPH in ovo.

## Figures and Tables

**Table 1:** Genes included in the Nanostring panel used for analysis of transcriptomic changes in liver of hatchling American kestrels (*Falco sparverius*) exposed to flame retardants in ovo.

Symbol	Gene Name	Function
*EEF1A*	Eukaryotic translation elongation factor 1 alpha 1	Housekeeping gene
*PPIA*	Peptidylprolyl isomerase A	Housekeeping gene
*TBP*	TATA box-binding protein	Housekeeping gene
*IFNB*	Interferon beta	Immune function
*IFNG*	Interferon gamma	Immune function
*IL18*	Interleukin 18	Immune function
*IL6*	Interleukin 6	Immune function
*IRF7*	IFN regulatory factor 7	Immune function
*RACK1*	Receptor for activated C kinase 1	Immune function
*RIGI*	Retinoic acid inducible gene I	Immune function
*STAT3*	Signal transducer and activator of transcription 3	Immune function
*TLR3*	Toll-like receptor 3	Immune function
*CEBPB*	CCAAT/enhancer-binding protein beta	Immune function
*CYP7B1*	Cytochrome P450, family 7, subfamily B, polypeptide 1	Lipid homeostasis
*FABP1*	Fatty acid-binding protein 1, liver	Lipid homeostasis
*FABP4*	Fatty acid-binding protein 4	Lipid homeostasis
*HMGCR*	3-Hydroxy-3-methylglutaryl-coenzyme A reductase	Lipid homeostasis
*LPL*	Lipoprotein lipase	Lipid homeostasis
*GPX1*	Glutathione peroxidase 1	Oxidative stress
*GSTA*	Glutathione *S*-transferase class-alpha	Oxidative stress
*SOD*	Superoxide dismutase	Oxidative stress
*PPARD*	Peroxisome proliferator-activated receptor delta	PPAR signaling pathway
*PPARA*	Peroxisome proliferator-activated receptor alpha	PPAR signaling pathway
*PPARG*	Peroxisome proliferator-activated receptor gamma	PPARsignaling pathway
*DIO1*	Iodothyronine deiodinase 1	Thyroid hormone pathway
*DIO2*	Iodothyronine deiodinase 2	Thyroid hormone pathway
*THRA*	Thyroid hormone receptor alpha	Thyroid hormone pathway
*THRB*	Thyroid hormone receptor beta	Thyroid hormone pathway
*TTR*	Transthyretin	Thyroid hormone pathway
*AHR*	Aryl hydrocarbon receptor	Xenobiotic metabolism
*CYP1A4*	Cytochrome P450 1A4	Xenobiotic metabolism
*CYP2H1*	cytochrome P450 2H1	Xenobiotic metabolism
*CYP3A37*	Cytochrome P450 A 37	Xenobiotic metabolism
*SULT1B1*	Sulfotransferase family, cytosolic, 1B, member 1	Xenobiotic metabolism

## Data Availability

Gene expression data generated during this study are available as a USGS data release [54]. The American kestrel de novo transcriptome used for probe design is available at https://www.ncbi.nlm.nih.gov/bioproject/?term=PRJNA313951 (accessed on 13 September 2018).

## Supplementary Materials

The following supporting information can be downloaded at https://www.mdpi.com/article/10.3390/biology11091341/s1: Table S1 (Relative mRNA expression profiles of 26 genes in female American kestrel hatchling livers) and Table S2 (Relative mRNA expression profiles of 26 genes in male American kestrel hatchling livers).

## References

[1] Renner R. (2004). In U.S., flame retardants will be voluntarily phased out. Environ. Sci. Technol..

[2] Stapleton H.M., Allen J.G., Kelly S.M., Konstantinov A., Klosterhaus S., Watkins D., McClean M.D., Webster T.F. (2008). Alternate and new brominated flame retardants detected in U.S. House Dust. Environ. Sci. Technol..

[3] Tongue A.D.W., Fernie K.J., Harrad S., Drage D.S., McGill R.A.R., Reynolds S.J. (2020). Interspecies comparisons of brominated flame retardants in relation to foraging ecology and behaviour of gulls frequenting a UK landfill. Sci. Total Environ..

[4] Tongue A.D.W., Reynolds S.J., Fernie K.J., Harrad S. (2019). Flame retardant concentrations and profiles in wild birds associated with landfill: A critical review. Environ. Pollut..

[5] Möller A., Xie Z., Cai M., Zhong G., Huang P., Cai M., Sturm R., He J., Ebinghaus R. (2011). Polybrominated Diphenyl Ethers vs. Alternate brominated flame retardants and dechloranes from east Asia to the arctic. Environ. Sci. Technol..

[6] Ma Y., Venier M., Hites R.A. (2012). 2-Ethylhexyl tetrabromobenzoate and Bis(2-ethylhexyl) tetrabromophthalate flame retardants in the great lakes atmosphere. Environ. Sci. Technol..

[7] Newton S., Sellström U., de Wit C.A. (2015). Emerging flame retardants, PBDEs, and HBCDDs in indoor and outdoor media in Stockholm, Sweden. Environ. Sci. Technol..

[8] Sagerup K., Herzke D., Harju M., Evenset A., Christensen G.N., Routti H., Fuglei E., Aars J., Strøm H., Gabrielsen G.W. (2010). New Brominated Flame Retardants in Arctic biota.

[9] Verreault J., Letcher R.J., Gentes M.L., Braune B.M. (2018). Unusually high Deca-BDE concentrations and new flame retardants in a Canadian Arctic top predator, the glaucous gull. Sci. Total Environ..

[10] Gentes M.L., Letcher R.J., Caron-Beaudoin É., Verreault J. (2012). Novel flame retardants in urban-feeding ring-billed gulls from the St. Lawrence River, Canada. Environ. Sci. Technol..

[11] Zhang X.L., Luo X.J., Liu H.Y., Yu L.H., Chen S.J., Mai B.X. (2011). Bioaccumulation of several brominated flame retardants and dechlorane plus in waterbirds from an e-waste recycling region in south China: Associated with trophic level and diet sources. Environ. Sci. Technol..

[12] Jin X., Lee S., Jeong Y., Yu J.P., Baek W.K., Shin K.H., Kannan K., Moon H.B. (2016). Species-specific accumulation of polybrominated diphenyl ethers (PBDEs) and other emerging flame retardants in several species of birds from Korea. Environ. Pollut..

[13] Sagerup K., Leonards P., Routti H., Fuglei E., Aars J., Strøm H., Kovacs K., Lydersen C., Gabrielsen G. (2011). Organophosphorus Flame Retardants in Arctic biota.

[14] Gauthier L.T., Hebert C.E., Weseloh D.V.C., Letcher R.J. (2007). Current-use flame retardants in the eggs of herring gulls (*Larus argentatus*) from the Laurentian Great Lakes. Environ. Sci. Technol..

[15] Gauthier L.T., Hebert C.E., Weseloh D.V.C., Letcher R.J. (2008). Dramatic changes in the temporal trends of polybrominated diphenyl ethers (PBDEs) in herring gull eggs from the Laurentian Great Lakes: 1982–2006. Environ. Sci. Technol..

[16] Karlsson M., Ericson I., van Bavel B., Jensen J.K., Dam M. (2006). Levels of brominated flame retardants in Northern Fulmar (*Fulmarus glacialis*) eggs from the Faroe Islands. Sci. Total Environ..

[17] Vorkamp K., Falk K., Møller S., Rigét F.F., Sørensen P.B. (2018). Regulated and unregulated halogenated flame retardants in peregrine falcon eggs from Greenland. Environ. Sci. Technol..

[18] Guerra P., Alaee M., Jiménez B., Pacepavicius G., Marvin C., MacInnis G., Eljarrat E., Barceló D., Champoux L., Fernie K. (2012). Emerging and historical brominated flame retardants in peregrine falcon (*Falco peregrinus*) eggs from Canada and Spain. Environ. Int..

[19] Fernie K.J., Chabot D., Champoux L., Brimble S., Alaee M., Marteinson S., Chen D., Palace V., Bird D.M., Letcher R.J. (2017). Spatiotemporal patterns and relationships among the diet, biochemistry, and exposure to flame retardants in an apex avian predator, the peregrine falcon. Environ. Res..

[20] Guo J., Simon K., Romanak K., Bowerman W., Venier M. (2018). Accumulation of flame retardants in paired eggs and plasma of bald eagles. Environ. Pollut..

[21] Bardo L., Bird D.M. (2009). The use of captive American kestrels (*Falco sparverius*) as wildlife models: A review. J. Raptor Res..

[22] Eng M.L., Karouna-Renier N.K., Henry P.F.P., Letcher R.J., Schultz S.L., Bean T.G., Peters L.E., Palace V.P., Williams T.D., Elliott J.E. (2019). *In ovo* exposure to brominated flame retardants Part II: Assessment of effects of TBBPA-BDBPE and BTBPE on hatching success, morphometric and physiological endpoints in American kestrels. Ecotoxicol. Environ. Saf..

[23] Goodchild C., Karouna-Renier N.K., Henry P.F.P., Letcher R.J., Schultz S.L., Maddox C.M., Bean T.G., Peters L.E., Palace V., Fernie K.J. (2021). Thyroid disruption and oxidative stress in American kestrels following embryonic exposure to the alternative flame retardants, EHTBB and TBPH. Environ. Int..

[24] Mingming T., Pu X., Xiaowei Z. (2022). Applications of functional genomics in uncovering the toxicity mechanisms of environmental chemicals. Asian J. Ecotoxicol..

[25] Crump D., Williams K.L., Chiu S., Letcher R.J., Periard L., Kennedy S.W. (2015). Biochemical and transcriptomic effects of herring gull egg extracts from variably contaminated colonies of the Laurentian Great Lakes in chicken hepatocytes. Environ. Sci. Technol..

[26] Klimstra J.D., Stebbins K.R., Heinz G.H., Hoffman D.J., Kondrad S.R. (2009). Factors related to the artificial incubation of wild bird eggs. Avian Biol. Res..

[27] Rattner B.A., Lazarus R.S., Heinz G.H., Karouna-Renier N.K., Schultz S.L., Hale R.C. (2013). Comparative embryotoxicity of a pentabrominated diphenyl etheer mixture to common terns (*Sterna hirundo)* and American kestrels (*Falco sparverius*). Chemosphere.

[28] Guigueno M.F., Karouna-Renier N.K., Henry P.F.P., Peters L.E., Palace V.P., Letcher R.J., Fernie K.J. (2018). Sex-specific responses in neuroanatomy of hatchling American kestrels in response to embryonic exposure to the flame retardants bis(2-ethylhexyl)-2,3,4,5-tetrabromophthalate and 2-ethylhexyl-2,3,4,5-tetrabromobenzoate. Environ. Toxicol. Chem..

[29] Wang H., Zhai T., Wang C. (2020). NanoStringDiff: Differential expression analysis of NanoString nCounter data. R Package Version.

[30] Smythe T.A., Butt C.M., Stapleton H.M., Pleskach K., Ratnayake G., Song C.Y., Riddell N., Konstantinov A., Tomy G.T. (2017). Impacts of unregulated novel brominated flame retardants on human liver thyroid deiodination and sulfotransferation. Environ. Sci. Technol..

[31] Hill K.L., Mortensen Å.-K., Teclechiel D., Willmore W.G., Sylte I., Jenssen B.M., Letcher R.J. (2018). In Vitro and in silico competitive binding of brominated polyphenyl ether contaminants with human and gull thyroid hormone transport proteins. Environ. Sci. Technol..

[32] Mortensen Å.-K., Mæhre S., Kristiansen K., Heimstad E.S., Gabrielsen G.W., Jenssen B.M., Sylte I. (2020). Homology modeling to screen for potential binding of contaminants to thyroid hormone receptor and transthyretin in glaucous gull (*Larus hyperboreus*) and herring gull (*Larus argentatus*). Comput. Toxicol..

[33] Egloff C., Crump D., Chiu S., Manning G., McLaren K.K., Cassone C.G., Letcher R.J., Gauthier L.T., Kennedy S.W. (2011). In vitro and *in ovo* effects of four brominated flame retardants on toxicity and hepatic mRNA expression in chicken embryos. Toxicol. Lett..

[34] Ourlin J.-C., Baader M., Fraser D., Halpert J.R., Meyer U.A. (2000). Cloning and functional expression of a first inducible avian cytochrome P450 of the CYP3A subfamily (CYP3A37). Arch. Biochem. Biophys..

[35] Watanabe K.P., Kawai Y.K., Ikenaka Y., Kawata M., Ikushiro S.I., Sakaki T., Ishizuka M. (2013). Avian cytochrome P450 (CYP) 1-3 family genes: Isoforms, evolutionary relationships, and mRNA expression in chicken liver. PLoS ONE.

[36] Barouki R., Morel Y. (2001). Repression of cytochrome P450 1A1 gene expression by oxidative stress: Mechanisms and biological implications. Biochem. Pharmacol..

[37] Regoli F., Giuliani M.E. (2014). Oxidative pathways of chemical toxicity and oxidative stress biomarkers in marine organisms. Mar. Environ. Res..

[38] Morel Y., Barouki R. (1998). Down-regulation of Cytochrome P450 1A1 Gene Promoter by Oxidative Stress: Critical Contribution of Nuclear Factor 1 *. J. Biol. Chem..

[39] Stavropoulou E., Pircalabioru G.G., Bezirtzoglou E. (2018). The Role of Cytochromes P450 in Infection. Front. Immunol..

[40] Porter E., Crump D., Egloff C., Chiu S., Kennedy S.W. (2014). Use of an avian hepatocyte assay and the avian toxchip polymerse chain reaction array for testing prioritization of 16 organic flame retardants. Environ. Toxicol. Chem..

[41] Farhat A., Crump D., Chiu S., Williams K.L., Letcher R.J., Gauthier L.T., Kennedy S.W. (2013). *In ovo* effects of two organophosphate flame retardants-TCPP and TDCPP-on pipping success, development, mRNA expression, and thyroid hormone levels in chicken embryos. Toxicol. Sci..

[42] He L., He T., Farrar S., Ji L., Liu T., Ma X. (2017). Antioxidants maintain cellular redox homeostasis by elimination of reactive oxygen species. Cell. Physiol. Biochem..

[43] Monaghan P., Metcalfe N.B., Torres R. (2009). Oxidative stress as a mediator of life history trade-offs: Mechanisms, measurements and interpretation. Ecol. Lett..

[44] Deeming D.C., Pike T.W. (2013). Embryonic growth and antioxidant provision in avian eggs. Biol. Lett..

[45] Chiang J.Y.L., Ferrell J.M. (2020). Up to date on cholesterol 7 alpha-hydroxylase (CYP7A1) in bile acid synthesis. Liver Res..

[46] Burg J.S., Espenshade P.J. (2011). Regulation of HMG-CoA reductase in mammals and yeast. Prog. Lipid Res..

[47] Crump D., Porter E., Egloff C., Williams K.L., Letcher R.J., Gauthier L.T., Kennedy S.W. (2014). 1,2-Dibromo-4-(1,2-dibromoethyl)-cyclohexane and tris(methylphenyl) phosphate cause significant effects on development, mRNA expression, and circulating bile acid concentrations in chicken embryos. Toxicol. Appl. Pharmacol..

[48] Wang Y., Nakajima T., Gonzalez F.J., Tanaka N. (2020). PPARs as metabolic regulators in the liver: Lessons from liver-specific PPAR-null mice. Int. J. Mol. Sci..

[49] Ali S.A.-F., Ismail A.A., Abdel-Hafez S.A., El-Genaidy H.M.A. (2020). Influence of thermally oxidized palm oil on growth performance and PPAR-α gene expression in broiler chickens. Egypt. Acad. J. Biol. Sciences. C Physiol. Mol. Biol..

[50] Parada R., Malewski T., Jaszczak K., Kawka M. (2018). Alternative transcription of peroxisome proliferator-activated receptor gamma in the liver is associated with fatness of chickens. Braz. J. Poult. Sci..

[51] Guo W., Lei L., Shi X., Li R., Wang Q., Han J., Yang L., Chen L., Zhou B. (2021). Nonalcoholic fatty liver disease development in zebrafish upon exposure to bis(2-ethylhexyl)-2,3,4,5-tetrabromophthalate, a novel brominated flame retardant. Environ. Sci. Technol..

[52] Crump D., Egloff C., Chiu S., Letcher R.J., Chu S., Kennedy S.W. (2010). Pipping success, isomer-specific accumulation, and hepatic mRNA expression in chicken embryos exposed to HBCD. Toxicol. Sci..

[53] Giraudo M., Douville M., Letcher R.J., Houde M. (2017). Effects of food-borne exposure of juvenile rainbow trout (*Oncorhynchus mykiss*) to emerging brominated flame retardants 1,2-bis(2,4,6-tribromophenoxy)ethane and 2-ethylhexyl-2,3,4,5-tetrabromobenzoate. Aquat. Toxicol..

[54] Karouna-Renier N.K., Braham R.P. (2022). Hepatic gene expression changes in American kestrel hatchlings. USGS Data Release.

