# Supplementary material for: Hepatic Gene Expression Profiling of American Kestrels (Falco sparverius) Exposed In Ovo to Three Alternative Brominated Flame Retardants
Source: Biology (Basel). 2022 Sep 12;11(9):1341. doi: 10.3390/biology11091341 (PMC9495696; doi:10.3390/biology11091341)
Supplement: Supplementary file 1 [file biology-11-01341-s001.zip › biology-1851045-supplementary.pdf]

Gene	Female						Female						Female						Function
	10 ng/g EHTBB		50 ng/g EHTBB		100 ng/g EHTBB		10 ng/g TBPH		50 ng/g TBPH		100 ng/g TBPH		10 ng/g BTBPE		50 ng/g BTBPE		100 ng/g BTBPE		
	logFC	*p*	*logFC*	*p*	*logFC*	*p*	*logFC*	*p*	*logFC*	*p*	*logFC*	*p*	*logFC*	*p*	*logFC*	*p*	*logFC*	*p*	
IL18	0.11	0.53	-0.17	0.37	0.11	0.58	-0.04	0.84	-0.05	0.79	0.31	0.07	-0.02	0.93	0.04	0.88	0.43	0.10	Immune function
IRF7	-0.45	0.32	-0.10	0.83	0.71	0.15	-0.34	0.49	0.11	0.82	0.10	0.83	0.09	0.82	-0.12	0.76	-0.58	0.16	Immune function
RACK1	-0.20	0.18	-0.32	0.04	-0.07	0.68	-0.03	0.83	-0.04	0.76	-0.12	0.39	-0.12	0.26	-0.18	0.08	0.08	0.46	Immune function
STAT3	-0.09	0.69	-0.48	0.05	-0.03	0.90	0.11	0.63	-0.13	0.57	-0.29	0.21	0.13	0.55	-0.37	0.10	-0.40	0.07	Immune function
TLR3	0.12	0.66	-0.19	0.51	0.64	0.03	-0.04	0.87	0.12	0.61	0.18	0.43	0.23	0.33	-0.05	0.82	0.60	**0.01**	Immune function
CEBPB	-0.48	0.18	-0.91	0.02	-0.59	0.15	-0.22	0.44	-0.30	0.29	-0.76	0.01	-0.44	0.16	-0.78	0.01	-0.26	0.40	Immune function
CYP7B1	0.33	0.27	-0.34	0.28	-0.21	0.54	0.48	0.19	0.14	0.71	0.03	0.93	-0.06	0.86	-0.26	0.41	-0.03	0.92	Lipid homeostasis
FABP1	0.13	0.81	-0.68	0.25	1.31	0.03	-1.00	0.05	-0.54	0.29	-0.55	0.28	-0.50	0.32	-0.50	0.32	-1.57	**0.00**	Lipid homeostasis
HMGCR	0.16	0.44	0.01	0.98	0.07	0.76	0.03	0.88	0.09	0.63	0.07	0.72	0.02	0.92	-0.18	0.37	0.30	0.14	Lipid homeostasis
LPL	-0.26	0.40	-0.02	0.96	-0.13	0.72	-0.70	0.02	0.02	0.95	0.10	0.73	-0.06	0.84	-0.09	0.77	-0.14	0.65	Lipid homeostasis
GPX1	-0.28	0.19	-0.33	0.15	-0.02	0.95	-0.07	0.72	0.12	0.54	-0.09	0.66	-0.09	0.63	-0.37	0.04	0.11	0.53	Oxidative Stress
GSTA	0.30	0.24	0.03	0.92	0.49	0.08	-0.20	0.38	-0.26	0.26	0.04	0.87	0.14	0.57	-0.58	0.02	0.03	0.91	Oxidative Stress
SOD	0.17	0.37	0.28	0.14	0.22	0.27	0.02	0.89	0.02	0.89	0.29	0.08	0.24	0.21	0.21	0.25	0.36	0.05	Oxidative Stress
PPARA	0.07	0.82	-0.14	0.65	-0.13	0.71	-0.20	0.44	-0.05	0.84	-0.26	0.32	-0.03	0.92	-0.11	0.67	0.02	0.93	PPAR signaling pathway
PPARD	0.08	0.76	-0.31	0.25	-0.21	0.48	0.07	0.76	-0.05	0.84	-0.35	0.14	-0.06	0.78	-0.17	0.45	0.00	0.99	PPAR signaling pathway
PPARG	0.33	0.46	-0.26	0.58	-0.67	0.21	-0.53	0.14	-0.41	0.24	-0.04	0.91	-0.32	0.44	0.03	0.93	-0.27	0.51	PPAR signaling pathway
DIO1	-0.03	0.92	-0.15	0.58	-0.05	0.85	-0.31	0.24	0.29	0.29	-0.26	0.34	0.04	0.86	-0.30	0.16	-0.32	0.13	Thyroid Hormone pathway
DIO2	-0.77	0.02	-0.76	0.03	-0.51	0.16	-1.01	0.01	-0.07	0.85	-0.43	0.24	-0.73	0.03	-0.06	0.87	-0.54	0.11	Thyroid Hormone pathway
THRA	-0.03	0.91	0.05	0.85	-0.08	0.75	-0.23	0.19	0.04	0.81	-0.02	0.90	0.13	0.51	-0.03	0.90	0.06	0.74	Thyroid Hormone pathway
THRB	-0.17	0.34	-0.13	0.50	-0.04	0.85	-0.15	0.38	0.16	0.33	-0.11	0.52	0.16	0.30	-0.12	0.43	0.00	0.99	Thyroid Hormone pathway
TTR	0.27	0.44	0.07	0.86	0.27	0.48	-0.41	0.18	0.07	0.81	0.42	0.17	0.23	0.48	-0.05	0.89	0.07	0.84	Thyroid Hormone pathway
AHR	-0.25	0.38	-0.30	0.30	-0.22	0.48	-0.69	0.05	-0.18	0.61	-0.32	0.36	-0.33	0.23	-0.38	0.17	-0.69	**0.01**	Xenobiotic metabolism
CYP1A4	-0.27	0.37	-0.39	0.22	-0.26	0.45	0.14	0.74	-0.12	0.78	-0.18	0.66	-0.69	0.03	-0.48	0.14	-0.26	0.42	Xenobiotic metabolism
CYP2H1	0.18	0.42	0.12	0.62	-0.15	0.55	-0.19	0.45	-0.06	0.83	0.10	0.69	-0.05	0.84	0.20	0.43	0.17	0.50	Xenobiotic metabolism
CYP3A37	0.41	0.57	-0.59	0.44	-1.16	0.17	0.15	0.84	-0.47	0.54	-0.29	0.70	-0.26	0.71	0.55	0.44	0.16	0.82	Xenobiotic metabolism
SULT1B1	-0.04	0.83	-0.35	0.09	-0.23	0.29	-0.17	0.42	-0.30	0.16	-0.04	0.85	-0.15	0.43	-0.44	0.02	-0.04	0.83	Xenobiotic metabolism

Table S1. Relative mRNA expression profiles of 26 genes in female American kestrel hatchling livers analyzed using a Nanostring codeset following in ovo exposure to 10, 50, and 100 ng/g of the flame retardants EHTBB, TBPH, and BTBPE. The mean log_2 fold change for each dose group and corresponding *p*-value as determined using a generalized linear model (GLM) are provided for each gene. The *p*-values in bold met the false discovery rate (FDR) threshold of 0.1.

Gene	Male						Male						Male						Function
	10 ng/g EHTBB		50 ng/g EHTBB		100 ng/g EHTBB		10 ng/g TBPH		50 ng/g TBPH		100 ng/g TBPH		10 ng/g BTBPE		50 ng/g BTBPE		100 ng/g BTBPE		
	logFC	*p*	*logFC*	*p*	*logFC*	*p*	*logFC*	*p*	*logFC*	*p*	*logFC*	*p*	*logFC*	*p*	*logFC*	*p*	*logFC*	*p*	
IL18	0.05	0.80	-0.02	0.93	0.07	0.72	-0.16	0.43	0.04	0.84	-0.17	0.40	0.25	0.26	-0.15	0.51	-0.14	0.56	Immune function
IRF7	1.20	0.01	-0.59	0.24	-0.61	0.20	-0.09	0.81	0.23	0.53	0.08	0.84	0.44	0.28	-0.38	0.38	-0.23	0.60	Immune function
RACK1	-0.14	0.17	-0.04	0.71	0.00	1.00	0.01	0.93	0.03	0.80	-0.06	0.56	0.02	0.85	0.07	0.57	-0.22	0.07	Immune function
STAT3	0.04	0.84	0.22	0.27	0.01	0.96	0.13	0.49	-0.08	0.67	0.16	0.38	0.20	0.35	0.21	0.33	-0.01	0.95	Immune function
TLR3	-0.07	0.79	-0.09	0.75	0.08	0.77	-0.08	0.75	0.19	0.45	-0.03	0.89	0.37	0.10	0.08	0.72	0.00	0.99	Immune function
CEBPB	-0.31	0.50	-0.01	0.99	0.20	0.67	-0.35	0.25	-0.26	0.38	-0.41	0.17	0.04	0.91	-0.05	0.88	-0.56	0.12	Immune function
CYP7B1	0.26	0.32	0.14	0.61	0.49	0.06	0.86	**0.01**	0.36	0.26	0.39	0.22	0.63	0.12	0.53	0.19	1.21	**0.00**	Lipid homeostasis
FABP1	0.31	0.64	0.04	0.96	0.07	0.91	-0.38	0.51	-0.15	0.79	0.66	0.25	0.65	0.27	0.28	0.63	-0.15	0.80	Lipid homeostasis
HMGCR	-0.53	0.02	-0.18	0.43	-0.21	0.35	-0.25	0.18	-0.26	0.16	-0.24	0.19	-0.09	0.71	-0.27	0.27	-0.41	0.11	Lipid homeostasis
LPL	-0.16	0.66	0.16	0.69	0.03	0.95	0.23	0.45	0.24	0.43	0.31	0.32	0.44	0.19	0.32	0.35	0.26	0.47	Lipid homeostasis
GPx1	0.14	0.47	0.11	0.59	0.18	0.34	0.22	0.26	0.21	0.29	0.02	0.91	0.30	0.25	0.22	0.39	-0.07	0.80	Oxidative Stress
GSTA	0.09	0.76	-0.34	0.27	-0.10	0.73	-0.12	0.67	-0.29	0.29	-0.04	0.88	0.11	0.69	-0.12	0.66	-0.12	0.67	Oxidative Stress
SOD	0.03	0.84	0.06	0.75	0.09	0.58	0.27	0.13	0.17	0.34	0.24	0.18	0.09	0.63	0.05	0.81	0.04	0.83	Oxidative Stress
PPARA	-0.63	0.00	-0.54	0.02	-0.36	0.11	-0.65	**0.00**	-0.36	0.09	-0.65	**0.00**	-0.55	0.06	-0.36	0.21	-0.54	0.08	PPAR signaling pathway
PPARD	0.06	0.81	0.01	0.98	0.13	0.62	-0.03	0.91	-0.01	0.96	0.00	0.99	0.09	0.69	0.03	0.91	-0.14	0.56	PPAR signaling pathway
PPARG	-0.95	0.06	-0.43	0.41	-0.50	0.31	-0.68	0.08	-0.78	0.04	-0.76	0.05	-0.30	0.48	-0.37	0.40	-0.64	0.16	PPAR signaling pathway
DIO1	0.25	0.44	0.51	0.13	0.38	0.24	0.24	0.41	0.32	0.27	0.33	0.25	0.39	0.13	0.63	0.02	0.48	0.07	Thyroid Hormone pathway
DIO2	0.19	0.72	0.74	0.20	0.94	0.09	0.55	0.18	0.01	0.97	0.16	0.69	0.78	0.05	0.24	0.54	0.22	0.60	Thyroid Hormone pathway
THRA	0.03	0.86	-0.06	0.71	-0.14	0.38	-0.18	0.34	-0.10	0.59	0.04	0.82	-0.10	0.61	-0.17	0.41	-0.21	0.33	Thyroid Hormone pathway
THRB	-0.07	0.71	-0.13	0.50	-0.02	0.89	-0.19	0.28	-0.02	0.90	-0.12	0.51	0.01	0.94	0.15	0.41	-0.08	0.68	Thyroid Hormone pathway
TTR	0.16	0.56	0.20	0.50	0.41	0.15	0.54	0.02	0.32	0.16	0.53	0.02	0.75	0.04	0.44	0.23	-0.15	0.70	Thyroid Hormone pathway
AHR	0.21	0.56	0.51	0.17	0.19	0.60	-0.06	0.82	0.36	0.15	0.56	0.03	0.47	0.13	0.60	0.05	0.35	0.27	Xenobiotic metabolism
CYP1A4	0.30	0.43	0.13	0.74	0.09	0.80	0.33	0.33	0.02	0.96	-0.09	0.79	0.44	0.14	0.37	0.22	-0.09	0.76	Xenobiotic metabolism
CYP2H1	0.09	0.79	-0.03	0.94	0.08	0.80	0.28	0.39	-0.25	0.46	0.15	0.65	-0.06	0.86	0.07	0.82	0.08	0.80	Xenobiotic metabolism
CYP3A37	-0.52	0.37	-1.23	0.05	-0.42	0.47	-0.71	0.21	-1.02	0.07	-1.94	**0.00**	-1.10	0.06	-0.98	0.10	-1.48	0.02	Xenobiotic metabolism
SULT1B1	-0.21	0.10	0.01	0.94	-0.07	0.58	0.07	0.72	-0.02	0.91	0.21	0.30	-0.05	0.84	0.14	0.55	-0.20	0.44	Xenobiotic metabolism

Table S2. Relative mRNA expression profiles of 26 genes in male American kestrel hatchling livers analyzed using a Nanostring codeset following in ovo exposure to 10, 50, and 100 ng/g of the flame retardants EHTBB, TBPH, and BTBPE. The mean $\log_2$ fold change for each dose group and corresponding *p*-value as determined using a generalized linear model (GLM) are provided for each gene. The *p*-values in bold met the false discovery rate (FDR) threshold of 0.1.
